# A Case Report on Rabeprazole Desensitization for a Patient With Barrett’s Esophagus and Anaphylaxis to Multiple Proton Pump Inhibitors

**DOI:** 10.7759/cureus.77966

**Published:** 2025-01-25

**Authors:** Nana Chkhikvadze, Bidzina Kulumbegov, Sopo Kipshidze, Maia Gotua

**Affiliations:** 1 Allergy and Immunology, Center of Allergy and Immunology, Tbilisi, GEO; 2 Allergy and Immunology, Caucasus Medical Center, Tbilisi, GEO

**Keywords:** allergy and anaphylaxis, drug allergy, drug induced anaphylaxis, ppi allergy, ppi-induced, ppi induced anaphylaxis

## Abstract

We report the case of a 58-year-old male patient with Barrett’s esophagus who experienced anaphylaxis following the administration of omeprazole and esomeprazole. The patient was successfully desensitized using rabeprazole despite also being sensitized to rabeprazole.

Approximately seven years prior, the patient developed generalized urticaria, angioedema, respiratory distress, and loss of consciousness after taking omeprazole, necessitating emergency medical intervention. One year ago, the patient underwent a drug allergy workup with 20 mg of esomeprazole under our supervision at the clinic. As the prick test was negative, oral provocation with 1/64 of the full dose of esomeprazole (0.31 mg) was performed. However, a couple of hours after administration, the patient developed angioedema, urticaria, and shortness of breath, which led to hospitalization.

Given the clinical necessity for proton pump inhibitor (PPI) therapy due to Barrett's esophagus, as recommended by a gastroenterologist, desensitization to rabeprazole 20 mg was initiated in a hospital setting, adhering to established international protocols. Prior to the desensitization, the patient’s sensitivity to rabeprazole was confirmed through skin tests. The desensitization procedure was successful.

In conclusion, while omeprazole and esomeprazole have a high potential for cross-reactivity due to their structural similarity, rabeprazole may be a viable alternative, though hypersensitivity to rabeprazole should not be ruled out. This case highlights the necessity of considering cross-reactivity in PPI hypersensitivity and underscores the utility of skin tests prior to provocation. When no alternative PPI treatment options are available, a PPI desensitization protocol may be successfully implemented.

## Introduction

Proton pump inhibitors (PPIs) are widely prescribed for the treatment of gastrointestinal disorders, including gastroesophageal reflux disease (GERD), peptic ulcer disease, Helicobacter pylori infections, and eosinophilic esophagitis. They are also used to prevent gastric damage resulting from prolonged use of corticosteroids and nonsteroidal anti-inflammatory drugs (NSAIDs). These medications are generally well-tolerated and are associated with a low incidence of adverse effects, with a risk of side effects of approximately 1% to 3% [1,2]. However, drug hypersensitivity reactions (DHRs) related to PPIs are increasingly contributing to the number of referrals to allergy departments worldwide. Extensive cross-reactivity exists among PPIs, which can initially be assessed through skin testing and subsequently confirmed by drug provocation testing (DPT). Reports indicate a 61.6% cross-reactivity with another PPI and an 8.9% cross-reactivity with all PPIs in cases of immediate hypersensitivity reactions (HSRs), confirmed by skin tests or DPT. Immediate HSRs to PPIs can range from mild to severe and typically occur within hours of the last dose. Anaphylaxis accounts for 9.1% to 69.0% of cases while urticaria and/or angioedema occur in 26.2% to 90.9% of cases [2,3].

The management of patients with confirmed hypersensitivity to PPIs, including rabeprazole, presents a significant challenge due to the high rate of potential cross-reactivity and the limited availability of alternative medications with comparable efficacy and safety profiles [4]. Desensitization protocols offer a viable solution, allowing patients to continue essential treatments despite their hypersensitivity [3,5]. Nevertheless, documented cases of successful rabeprazole desensitization are sparse, creating a substantial gap in the clinical literature and guidelines for practitioners.

This clinical case report aims to address this gap by presenting our experience with rabeprazole desensitization in a patient with a documented hypersensitivity reaction. Through this report, we seek to contribute our practical guidance to the medical community highlighting the feasibility and safety of desensitization protocols in the management of PPI hypersensitivity. The significance of our findings lies not only in the successful outcome but also in providing a documented reference for clinicians encountering similar challenges.

## Case presentation

A 58-year-old male patient was diagnosed with Barrett's esophagus and was prescribed a PPI by his gastroenterologist. Seven years prior, the patient developed anaphylaxis (generalized urticaria, angioedema, hypotension, respiratory distress, and loss of consciousness) after taking omeprazole, necessitating emergency medical intervention. Due to the history of severe hypersensitivity, the patient was referred for a drug allergy workup. During the patient's history taking, no clinical signs of atopy or other allergies were detected.

As a significant amount of time has passed since the initial episode, which was based solely on the patient's account, one year ago, we decided to perform an oral graded challenge with a 20 mg dose of esomeprazole in the clinic under our supervision. The patient was administered a 1/64 dose (0.31 mg) of the drug. After one hour of observation with no reported symptoms, the procedure was scheduled to continue the following day. However, several hours later, the patient developed angioedema, urticaria, dyspnea, and hypotension, necessitating hospitalization.

Skin tests

Given the hypersensitivity reactions observed with both omeprazole and esomeprazole, suggesting possible cross-reactivity, skin testing with rabeprazole was planned. A skin prick test (SPT) was conducted using commercial oral preparations of rabeprazole tablet (20 mg), with histamine 0.1% and saline 0.9% used as positive and negative controls, respectively. For the SPT, the tablets were ground into a fine powder, and the solution was prepared by mixing with 1 mL of 0.9% saline. The SPT was performed on the forearm and read after 15 minutes. A positive result was defined as a wheal approximately 3 mm larger than the negative control site, accompanied by surrounding erythema. The rabeprazole SPT revealed a 4x4 mm papule (Table 1), indicating a positive hypersensitivity reaction.

**Table 1:** Skin test results with rabeprazole *(-): Negative

Drug	Before desensitization	After desensitization
Rabeprazole (20 mg/mL)	4x4 mm wheal/5x8 mm flare diameter	-/-
Negative control	(-)	(-)

Desensitization

The desensitization protocol was carried out in the allergy unit of the hospital under the supervision of nurses and doctors, after obtaining the consent of the patient. Resuscitation equipment was made available during the process. A rabeprazole 20 mg tablet was dissolved in 0.9% NaCl solution by creating serial 10-fold dilutions. In oral desensitization, the initial dose was 0.01 mg and the doses, which were increased twofold 12 times, were administered at 30-minute intervals (Table 2). The patient tolerated the procedure with no problems.

**Table 2:** Desensitization protocol with rabeprazole

Step	Dilution	Time (min)	Dose (ml)	Dose (mg)	Cumulative dose (mg)
1	1/1000	30	0.5	0.01	0.01
2	1/1000	30	1	0.02	0.03
3	1/1000	30	2	0.04	0.07
4	1/1000	30	4	0.08	0.15
5	1/100	30	0.5	0.1	0.25
6	1/100	30	1	0.2	0.45
7	1/100	30	2	0.4	0.85
8	1/100	30	4	0.8	1.65
9	1/10	30	0.5	1	2.65
10	1/10	30	1	3	5.65
11	1/10	30	3	6	11.65
12	1/10	30	6	12	23.65

The SPT performed with rabeprazole 24 hours after the completion of desensitization was negative. The patient subsequently tolerated a full 20 mg dose of rabeprazole, administered as two 10 mg doses with a 30-minute interval between them, followed by a final 1-hour observation period, with no adverse reactions observed. According to the gastroenterologist's prescription, the patient is now receiving a fourfold dose of rabeprazole.

## Discussion

The presented case underscores the complexity and importance of managing DHRs to PPIs, particularly in patients requiring continued therapy for conditions such as Barrett's esophagus [1,6,7]. In this case, the patient experienced anaphylaxis and severe hypersensitivity symptoms, including generalized urticaria, angioedema, hypotension, and respiratory distress, following exposure to omeprazole and esomeprazole, necessitating urgent medical intervention. This highlights the critical need for alternative treatment strategies when traditional PPIs provoke adverse reactions.

Managing PPI hypersensitivity, especially in cases involving multiple drug sensitivities, is challenging due to the structural similarities among PPIs and their high potential for cross-reactivity. Despite hypersensitivity to omeprazole and esomeprazole, this patient successfully underwent desensitization to rabeprazole, underscoring the importance of assessing cross-reactivity when devising alternative treatment strategies. Our findings align with reports of successful desensitization protocols for PPIs in similar cases [3-5,8].

The subsequent oral desensitization protocol with rabeprazole was meticulously designed, employing serial dilutions to gradually increase the dose over several hours. This approach enabled the patient to tolerate a therapeutic dose of rabeprazole without adverse effects, demonstrating the efficacy and safety of the desensitization process.

It is also noteworthy that most PPIs are formulated as slow-release medications, which complicates dose titration and may contribute to delayed hypersensitivity reactions. This challenge necessitates extended observation periods following drug provocation testing (DPT) to monitor for delayed-onset reactions. The successful outcome in this case underscores the importance and feasibility of implementing prolonged observation protocols post-DPT to ensure patient safety and effective management of PPI hypersensitivity [2,9].

In a multicenter study, Bonadonna et al. demonstrated that skin tests (SPTs and IDTs) effectively diagnose PPI hypersensitivity with high specificity (100%) and positive predictive value (100%), though sensitivity was moderate (61.3%). Cross-reactivity was observed among structurally similar PPIs while PPIs with different side chains (e.g., rabeprazole, lansoprazole) showed less. Positive skin tests may reduce the need for riskier oral provocation testing, though further research is needed [10].

Successful desensitization in this case demonstrates the potential of such protocols for managing PPI hypersensitivity. However, further research and standardized guidelines are needed to optimize these protocols. Variability in patient responses highlights the importance of individualized treatment, careful monitoring, and understanding PPI cross-reactivity mechanisms. Systematic approaches like skin testing, desensitization, and personalized care offer effective strategies, but further studies are essential to refine clinical management [1,4,7,9].

## Conclusions

In conclusion, while omeprazole and esomeprazole have a high potential for cross-reactivity due to their structural similarity, rabeprazole may be a viable alternative, though hypersensitivity to rabeprazole should not be ruled out. This underscores the importance of individualized approaches and prolonged observation in managing PPI hypersensitivity, ensuring safe and effective treatment for patients with complex medication allergies.
